# Images in Infectious Diseases in Obstetrics and Gynecology

**DOI:** 10.1155/S1064744996000129

**Published:** 1996

**Authors:** David E. Soper


					Infectious Diseases in Obstetrics and Gynecology 4:61 (1996)
(C) 1996 Wi!ey-Liss, Inc.
Images in Infectious Diseases in
Obstetrics and Gynecology
A picture is worth a thousand words. I think we all ascribe to this dictum. For this
reason, Infectious Diseases in ObstetHcs and Gynecology will begin a new section in which
clinically important visual images will be reported to allow its readers to appreciate
the visual nature ofthe broad spectrum ofinfectious diseases in obstetrics and gynecol-
ogy and in women's health care.
The section can offer insight regarding the pathophysiology of disease, as is pre-
sented in our first "Images" on pelvic inflammatory disease. It may also reflect
common disease states that we see weekly in our clinical practices or manifestations
of severe disease that we hope to see infrequently, as is planned in an upcoming
"Images" on necrotizing fasciitis.
We are hopeful that this section will become popular and highly anticipated, which
can only be accomplished with the help ofreaders willing to submit clinically important
visual images that a practitioner in women's health might encounter. Readers are
requested to submit high-quality color or black-and-white photographs or slides repre-
senting such images along with descriptive legends to David E. Soper, M.D., Division
of Benign Gynecology, Medical University of South Carolina, 171 Ashley Avenue,
Charleston, SC 29401-2233.
"Images" is made possible through an educational grant from Pfizer, Inc.
David E. Soper
Section Editor, "Images"
Editorial
Received July 5, 1996
Accepted July 8, 1996

				

